# Serum Zinc Level Is Associated with Frailty in Chronic Liver Diseases

**DOI:** 10.3390/jcm9051570

**Published:** 2020-05-22

**Authors:** Hiroki Nishikawa, Kazunori Yoh, Hirayuki Enomoto, Yoshinori Iwata, Yoshiyuki Sakai, Kyohei Kishino, Yoshihiro Shimono, Naoto Ikeda, Tomoyuki Takashima, Nobuhiro Aizawa, Ryo Takata, Kunihiro Hasegawa, Takashi Koriyama, Yukihisa Yuri, Takashi Nishimura, Shuhei Nishiguchi, Hiroko Iijima

**Affiliations:** 1 Department of Internal Medicine, Division of Gastroenterology and Hepatology, Hyogo College of Medicine, Nishinomiya 663-8501, Japan; mm2wintwin@ybb.ne.jp (K.Y.); enomoto@hyo-med.ac.jp (H.E.); yo-iwata@hyo-med.ac.jp (Y.I.); sakai429@hyo-med.ac.jp (Y.S.); hcm.kyohei@gmail.com (K.K.); yoshihiro19870729@yahoo.co.jp (Y.S.); nikeneko@hyo-med.ac.jp (N.I.); tomo0204@yahoo.co.jp (T.T.); nobu23hiro@yahoo.co.jp (N.A.); chano_chano_rt@yahoo.co.jp (R.T.); hiro.red1230@gmail.com (K.H.); takashi051114@yahoo.co.jp (T.K.); gyma27ijo04td@gmail.com (Y.Y.); tk-nishimura@hyo-med.ac.jp (T.N.); hiroko-i@hyo-med.ac.jp (H.I.); 2 Center for Clinical Research and Education, Hyogo College of Medicine, Nishinomiya 663-8501, Japan; 3 Kano General Hospital, Osaka 531-0041, Japan; shuhei813n@k.vodafone.ne.jp

**Keywords:** frailty, chronic liver disease, zinc, correlation

## Abstract

We sought to examine the serum zinc (Zn) level and frailty in patients with chronic liver diseases (CLDs, *n* = 285, 107 liver cirrhosis cases, median age = 66 years). Frailty was defined as a clinical syndrome in which three or more of the following criteria were met (frailty score 3, 4, or 5): unintentional body weight loss, self-reported exhaustion, muscle weakness (grip strength: <26 kg in men and <18 kg in women), slow walking speed (<1.0 m/s), and low physical activity. Robust (frailty score 0), prefrail (frailty score 1 or 2), and frailty were found in 90 (31.6%), 157 (55.1%), and 38 (13.3%), respectively. The median serum Zn levels in patients with frailty, prefrailty, and robust were 59.7 μg/dL, 72.8 μg/dL, and 76.9 μg/dL, respectively (*p*-values: frailty vs. prefrail, *p* < 0.0001; prefrail vs. robust, *p* = 0.0063; frailty vs. robust, *p* < 0.0001; overall *p* < 0.0001). For all cases, variables with absolute values of correlation coefficient with frailty score (0–5) ≥ 0.3 were age (*r_s_* = 0.3570, *p* < 0.0001), serum albumin (*r_s_* = −0.3212, *p* < 0.0001), extracellular water to total body water ratio using bioimpedance analysis (*r_s_* = 0.4386, *p* < 0.0001), and serum Zn level (*r_s_* = −0.3406, *p* < 0.0001). In conclusion, decreased serum Zn level in patients with CLDs can be closely associated with the presence of frailty.

## 1. Introduction

Frailty refers to a state in which vulnerability to physical stress is increased due to a decrease in physiological function, and it precedes disability [1,2,3]. In case of frailty, it is easy to fall into an outcome such as requiring nursing care or death [1,2,3]. Originally it was proposed to identify elderly people at elevated risk of poor health outcomes, dependencies, falls, disabilities, and mortality [1,2,3]. The Japan Geriatric Society defines frailty as an intermediate between health and need of care [4]. Frailty encompasses not only physical aspects but also social and mental aspects, capturing patients’ health in a broader category when compared to sarcopenia characterized by a progressive and generalized decline of skeletal muscle mass, muscle strength, and/or physical activity (PA) [2,3,4,5]. Frailty is defined as the presence of three or more of the following phenotypes: body weight (BW) loss, self-reported exhaustion, skeletal muscle functional decline, slow walking speed (WS), and decreased PA [4,5]. Recently, the concept of frailty has been enlarged to chronic liver diseases (CLDs) as clinical symptoms of impaired global physical function [6,7,8,9,10,11,12,13,14,15]. Tanaka et al. reported that frailty, age of 76 years or more, and open surgery were independent adverse predictors associated with loss of independence after surgery in independently living subjects aged 65 years or more undergoing liver surgery [6]. Carey et al. reported that 6 min walk distance reflecting physical function significantly predicted mortality in liver transplant candidates [7].

Zinc (Zn) is a component of hundreds of enzyme proteins that are involved in a variety of in vivo reactions [16,17,18]. Because Zn is required for the synthesis of proteins from amino acids and the synthesis of DNA, it is an essential trace element in tissues and organs where new cells are made [16,17,18]. Zn also plays a significant role for growth, wound healing, skin metabolism, immunity, and the maintenance of sensory organs [16,17,18,19,20,21,22,23]. Zn deficiency can therefore result in a large amount of clinical symptoms [16,17,18,19,20,21,22]. Many previous studies have shown that most CLD patients have Zn deficiency due to reduced Zn absorption from gastrointestinal tracts and increased urinary excretion of Zn [21,23,24,25]. Zn deficiency can be associated with numerous metabolic abnormalities in CLDs including insulin resistance, hepatic steatosis, and hepatic encephalopathy [23]. Conversely, metabolic disorders such as hypoalbuminemia in liver cirrhosis (LC) patients frequently result in Zn deficiency [23]. However, the relationship between the serum Zn level and frailty remains unclear in patients with CLDs. The aims of the study were to explore the relationship between the serum Zn level and frailty in CLD patients.

## 2. Patients and Methods

### 2.1. Patients 

A total of 285 CLD subjects with frailty assessment and serum Zn level consulted our hospital between July 2015 and March 2019, and they were subjected to this analysis. LC was determined by liver biopsy samples, radiological findings (deformation of the liver surface, varices or splenomegaly, etc.), liver fibrosis markers (e.g., FIB4 index > 3.25), and/or laboratory data (e.g., lower platelet count or prolonged prothrombin time) [26,27,28,29,30].

Frailty was defined as a clinical syndrome in which three or more of the following criteria were met (frailty score 3, 4, or 5): unintentional BW loss (2, 3 kg or more BW loss within the past 6 months), self-reported exhaustion, muscle weakness (grip strength (GS): <26 kg in men and <18 kg in women), slow WS (<1.0 m/s), and low PA (doing light exercise or not), whereas prefrail was defined as patients with one or two aforementioned phenotypes (frailty score 1 or 2). Patients with none of the five phenotypes were regarded as having robust status (frailty score 0) [4,5]. GS was measured according to the current guidelines [31]. Skeletal muscle index (SMI) was tested using bioimpedance analysis (BIA), as described previously [32]. In all analyzed subjects, a 6 m walking test was done. The 6 m walking test was done twice in all subjects, with WS (m/s) being defined as the mean value of them. Because of the intrinsic limitations of BIA, which is hampered by ascites, patients with severe ascites were not subject to this study. We investigated the relationship between the serum Zn level and frailty in CLD patients in a retrospective manner.

The institutional review board in our hospital acknowledged this research protocol, and the Declaration of Helsinki was strictly adhered to in order to ensure the rights of the patients. An opt-out approach was employed in order to obtain informed consent from the study subjects.

### 2.2. Statistical Considerations

The JMP 14 software (SAS Institute Inc., Cary, NC, USA) was used to for statistical analysis. For the numerical variables, Mann–Whitney *U* test, Student’s *t*-test, Spearman’s rank coefficient *r_s_*, analysis of variance, or Kruskal–Wallis test was used to assess group characteristics when appropriate. Variables with statistical significance for the correlation with frailty score for all cases were subjected to the multivariate regression analysis with multiple predictive variables using least squares method to select candidate variables. Numerical data were expressed as median value (interquartile range (IQR)). The statistical significant level was set at *p* < 0.05.

## 3. Results

### 3.1. Baseline Features

Table 1 summarized the baseline features of the study cohort (*n* = 285). The study cohort included 126 males and 159 females with the median age (IQR) of 66 (55.5, 72) years. There were 107 LC cases (37.5%). There were 75 patients with Child-Pugh A, 29 with Child-Pugh B, and 3 with Child -Pugh C. The median (IQR) serum Zn level was 73 (64.25, 82) μg/dL. The median (IQR) serum Zn level in patients with non-LC, and Child-Pugh A, B, and C were 77.1 (70.9, 84.6) μg/dL, 67.9 (60.5, 75.6) μg/dL, 49 (42.3, 57.1) μg/dL, and 29 (22, 37.6) μg/dL, respectively. In LC patients, Child-Pugh score had a significant inverse correlation with serum Zn level (*r_s_* = −0.6043, *p* < 0.0001). In men, the median (IQR) GS was 33.0 (27.25, 37.075) kg, whereas in women, the median (IQR) GS was 20.8 (17.7, 24.7) kg. A total of 27 men (21.4%) and 43 women (27.0%) had GS decrease (GS: <26 kg for men and <18 kg for women). The median (IQR) WS was 1.314 (1.111, 1.447) m/s. A total of 40 patients (14.0%) had WS decrease (<1.0 m/s), 139 patients (48.8%) reported exhaustion, 10 patients (3.5%) reported BW loss, and 76 patients (26.7%) reported low physical activity. Frailty score ranged from 0 to 5 (median, 1). Robust (frailty score 0), prefrail (frailty score 1 or 2), and frailty (frailty score 3 or more) were found in 90 (31.6%), 157 (55.1%), and 38 (13.3%) participants, respectively. Sarcopenia, as assessed by Japanese guidelines, was identified in 42 patients (14.7%) [31]. Of these, 15 patients had frailty, and the remaining 27 patients had prefrail.

### 3.2. Serum Zn Level in Patients with Frailty, Prefrail, and Robust

**Figure 1:**
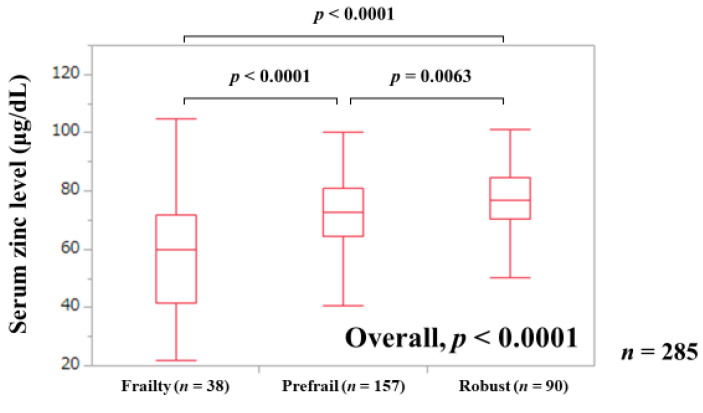
Serum Zn level according to the frailty status.

The median (IQR) serum Zn levels in patients with frailty (*n* = 38), prefrail (*n* = 157), and robust (*n* = 90) were 59.7 (41.625, 71.625) μg/dL, 72.8 (64.45, 81.1) μg/dL, and 76.9 (70.625, 84.85) μg/dL, respectively (*p*-values: frailty vs. prefrail, *p* < 0.0001; prefrail vs. robust, *p* = 0.0063; frailty vs. robust, *p* < 0.0001; overall *p* < 0.0001) (Figure 1). 

### 3.3. Correlation between Serum Zn Level and WS and GS

**Figure 2:**
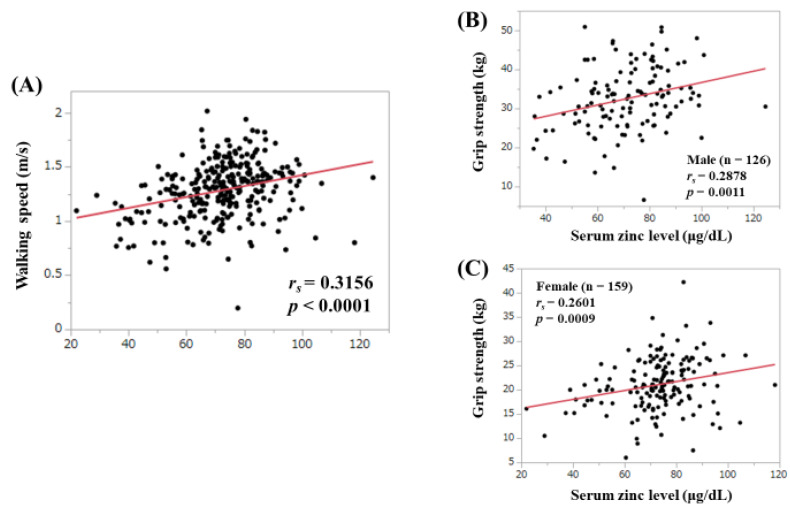
(**A**) Correlation between the serum Zn level and walking speed. (**B**) Correlation between the serum Zn level and grip strength in men. (**C**) Correlation between the serum Zn level and grip strength in women.

There was a significant correlation between serum Zn level and WS (*r_s_* = 0.3156, *p* < 0.0001). (Figure 2A). Correlation between serum Zn level and GS in males (*r_s_* = 0.2878, *p* = 0.0011) and females (*r_s_* = 0.2601, *p* = 0.0009) were also significant (Figure 2B,C). 

### 3.4. Serum Zn Level According to the Frailty Phenotypes

**Figure 3:**
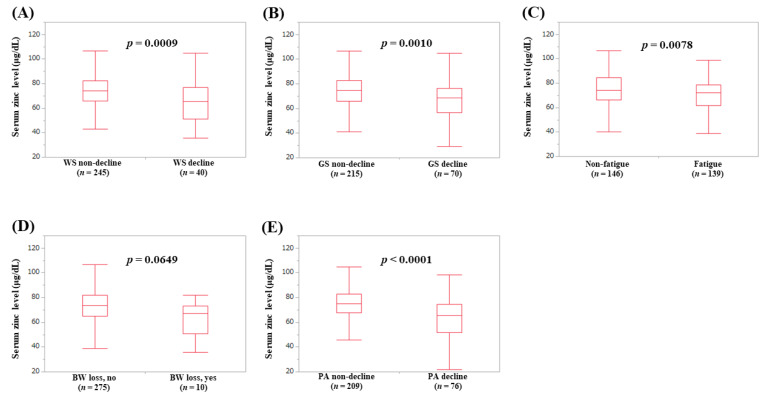
Serum Zn level according to each frailty phenotype: (**A**) walking speed, (**B**) grip strength, (**C**) fatigue, (**D**) body weight loss, (**E**) physical activity.

The median (IQR) serum Zn levels in patients with WS decline and without WS decline were 65.45 (51.25, 76.975) μg/dL and 74.2 (65.75, 82.2) μg/dL, respectively (*p* = 0.0009) (Figure 3A). The median (IQR) serum Zn levels in patients with GS decline and without GS decline were 68.5 (56.45, 76.525) μg/dL and 74.5 (65.9, 82.8) μg/dL, respectively (*p* = 0.0010) (Figure 3B). The median (IQR) serum Zn levels in patients with fatigue and without fatigue were 72.3 (61.5, 78.5) μg/dL and 74.1 (66.2, 84.625) μg/dL, respectively (*p* = 0.0078) (Figure 3C). The median (IQR) serum Zn levels in patients with BW loss and without BW loss were 67.3 (50.5, 73.3) μg/dL and 73.4 (64.7, 82.1) μg/dL, respectively (*p* = 0.0649) (Figure 3D). The median (IQR) serum Zn levels in patients with PA decline and without PA decline were 65.25 (51.8, 74.475) μg/dL and 75.1 (67.8, 82.85) μg/dL, respectively (*p* < 0.0001) (Figure 3E). 

### 3.5. Serum Zn Level According to the Frailty Phenotypes in LC Patients (Subset Analysis 1)

**Figure 4:**
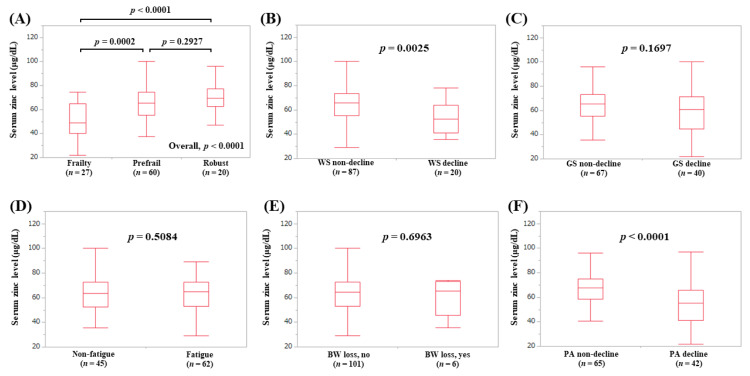
Serum Zn level according to the frailty status (**A**) and serum Zn level according to each frailty phenotype in LC patients: (**B**) walking speed, (**C**) grip strength, (**D**) fatigue, (**E**) body weight loss, (**F**) physical activity.

The median (IQR) serum Zn levels in LC patients with frailty (*n* = 27), prefrail (*n* = 60), and robust (*n* = 20) were 49 (40, 65) μg/dL, 65.4 (55.125, 74.6) μg/dL, and 69.55 (62.45, 77.3) μg/dL, respectively (*p*-values: frailty vs. prefrail, *p* = 0.0002; prefrail vs. robust, *p* = 0.2927; frailty vs. robust, *p* < 0.0001; overall *p* < 0.0001) (Figure 4A). In terms of frailty phenotypes, the serum Zn levels in LC patients with WS decline (*p* = 0.0025, Figure 4B) and PA decline (*p* < 0.0001, Figure 4F) were lower with statistical significance compared with each counterpart.

### 3.6. Serum Zn Level According to the Frailty Phenotypes in Non-LC Patients (Subset Analysis 2)

**Figure 5:**
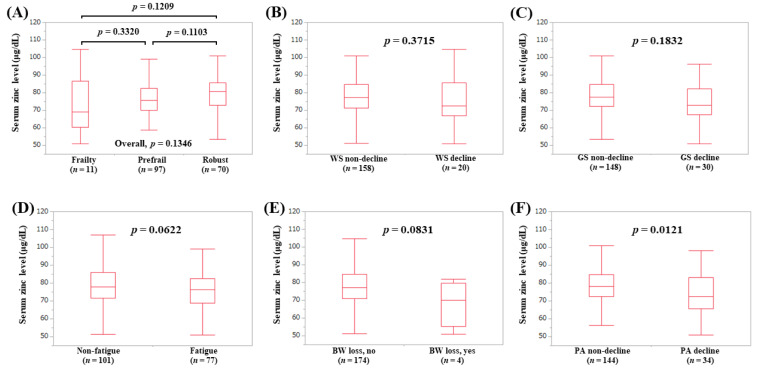
Serum Zn level according to the frailty status (**A**) and serum Zn level according to each frailty phenotype in non-LC patients: (**B**) walking speed, (**C**) grip strength, (**D**) fatigue, (**E**) body weight loss, (**F**) physical activity.

The median (IQR) serum Zn levels in non-LC patients with frailty (*n* = 11), prefrail (*n* = 97), and robust (*n* = 70) were 69.2 (60.3, 86.6) μg/dL, 75.5 (70.1, 82.5) μg/dL, and 80.65 (72.7, 85.775) μg/dL, respectively (*p*-values: frailty vs. prefrail, *p* = 0.3320; prefrail vs. robust, *p* = 0.1103; frailty vs. robust, *p* = 0.1209; overall *p* = 0.1346) (Figure 5A). In terms of frailty phenotypes, the serum Zn level in non-LC patients with PA decline (*p* = 0.0121, Figure 5F) was lower in terms of statistical significance compared with the counterpart.

### 3.7. Serum Zn Level According to the Frailty Phenotypes in Elderly Patients (65 Years or More, Subset Analysis 3)

**Figure 6:**
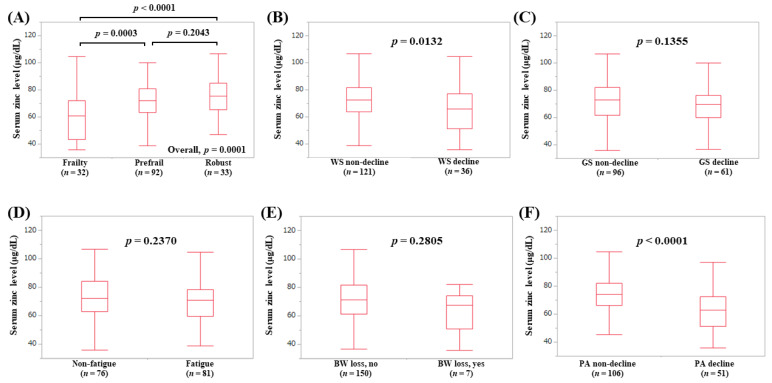
Serum Zn level according to the frailty status (**A**) and serum Zn level according to each frailty phenotype in patients aged 65 years or more: (**B**) walking speed, (**C**) grip strength, (**D**) fatigue, (**E**) body weight loss, (**F**) physical activity.

The median (IQR) serum Zn levels in elderly patients with frailty (*n* = 32), prefrail (*n* = 92), and robust (*n* = 33) were 60.95 (43.225, 72.075) μg/dL, 72.25 (63.225, 80.7) μg/dL, and 75.4 (65.3, 85.1) μg/dL, respectively (*p*-values: frailty vs. prefrail, *p* = 0.0003; prefrail vs. robust, *p* = 0.2043; frailty vs. robust, *p* < 0.0001; overall *p* = 0.0001) (Figure 6A). In terms of frailty phenotypes, the serum Zn levels in elderly patients with WS decline (*p* = 0.0132, Figure 6B) and PA decline (*p* < 0.0001, Figure 6F) were lower in terms of statistical significance compared with each counterpart.

### 3.8. Serum Zn Level According to the Frailty Phenotypes in Younger Patients (Less than 65 Years, Subset Analysis 4)

**Figure 7:**
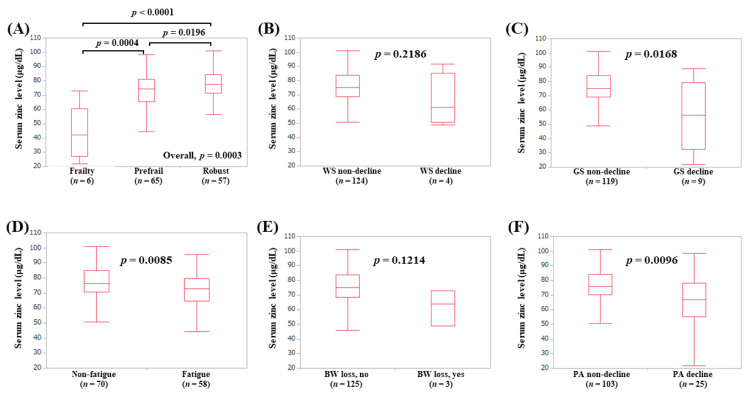
Serum Zn level according to the frailty status (**A**) and serum Zn level according to each frailty phenotype in patients less than 65 years: (**B**) walking speed, (**C**) grip strength, (**D**) fatigue, (**E**) body weight loss, (**F**) physical activity.

The median (IQR) serum Zn levels in younger patients with frailty (*n* = 6), prefrail (*n* = 65), and robust (*n* = 57) were 42.2 (27.25, 60.475) μg/dL, 74.5 (65.4, 81.2) μg/dL, and 77.3 (71.3, 84.65) μg/dL, respectively (*p*-values: frailty vs. prefrail, *p* = 0.0004; prefrail vs. robust, *p* = 0.0196; frailty vs. robust, *p* < 0.0001; overall *p* = 0.0003) (Figure 7A). In terms of frailty phenotypes, the serum Zn levels in elderly patients with GS decline (*p* = 0.0168, Figure 7C), fatigue (*p* = 0.0085, Figure 7D), and PA decline (*p* = 0.0096, Figure 7F) were lower in terms of statistical significance compared with each counterpart.

### 3.9. Serum Zn Level According to the Frailty Phenotypes in Male Patients (Subset Analysis 5)

**Figure 8:**
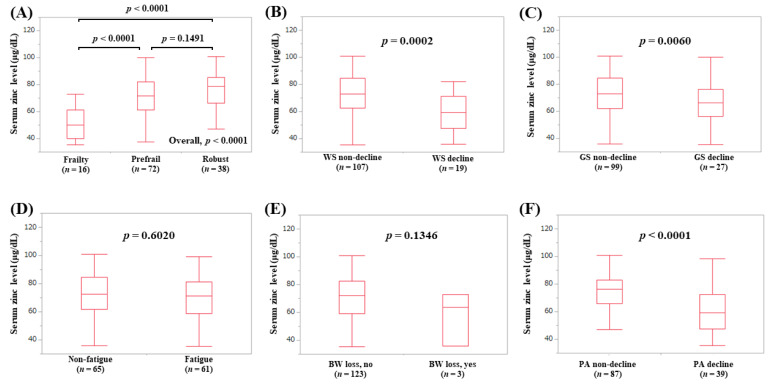
Serum Zn level according to the frailty status (**A**) and serum Zn level according to each frailty phenotype in male patients: (**B**) walking speed, (**C**) grip strength, (**D**) fatigue, (**E**) body weight loss, (**F**) physical activity.

The median (IQR) serum Zn levels in male patients with frailty (*n* = 16), prefrail (*n* = 72), and robust (*n* = 38) were 50.15 (40.075, 61.125) μg/dL, 71.7 (61.3, 82.05) μg/dL, and 78.95 (66.2, 85.4) μg/dL, respectively (*p*-values: frailty vs. prefrail, *p* < 0.0001; prefrail vs. robust, *p* = 0.1491; frailty vs. robust, *p* < 0.0001; overall *p* < 0.0001) (Figure 8A). In terms of frailty phenotypes, the serum Zn levels in male patients with WS decline (*p* = 0.0002, Figure 8B), GS decline (*p* = 0.0060, Figure 8C), and PA decline (*p* < 0.0001, Figure 8F) were lower in terms of statistical significance compared with each counterpart.

### 3.10. Serum Zn Level According to the Frailty Phenotypes in Female Patients (Subset Analysis 6)

**Figure 9:**
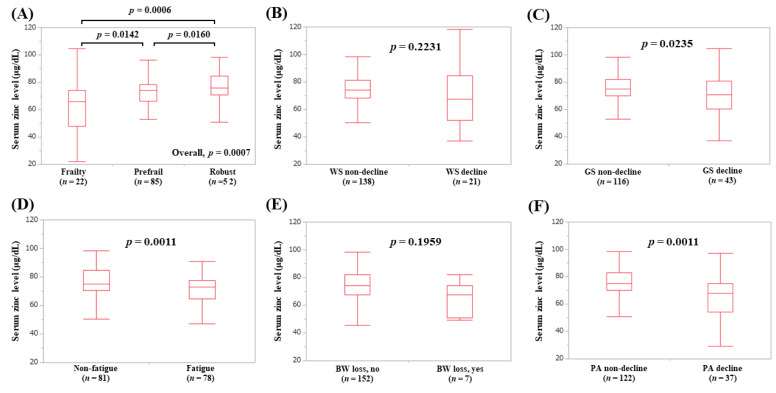
Serum Zn level according to the frailty status (**A**) and serum Zn level according to each frailty phenotype in female patients: (**B**) walking speed, (**C**) grip strength, (**D**) fatigue, (**E**) body weight loss, (**F**) physical activity.

The median (IQR) serum Zn levels in female patients with frailty (*n* = 22), prefrail (*n* = 85), and robust (*n* = 52) were 65.95 (47.875, 74.275) μg/dL, 74.4 (67.75, 80.2) μg/dL, and 75.8 (70.975, 84.65) μg/dL, respectively (*p*-values: frailty vs. prefrail, *p* = 0.0142; prefrail vs. robust, *p* = 0.0160; frailty vs. robust, *p* = 0.0006; overall *p* = 0.0007) (Figure 9A). In terms of frailty phenotypes, the serum Zn levels in female patients with GS decline (*p* = 0.0235, Figure 9C), fatigue (*p* = 0.0011, Figure 9D), and PA decline (*p* = 0.0011, Figure 9F) were lower in terms of statistical significance compared with each counterpart.

### 3.11. Correlation between Frailty Score (0–5) and Baseline Numerical Variables

Correlation between frailty score (0–5) and baseline numerical variables for all cases, male and female, are shown in Table 2. For all cases, variables with absolute values of correlation coefficient ≥0.3 were age (*r_s_* = 0.3570, *p* < 0.0001), serum albumin (*r_s_* = −0.3212, *p* < 0.0001), extracellular water (ECW) to total body water (TBW) ratio (*r_s_* = 0.4386, *p* < 0.0001), and serum Zn level (*r_s_* = −0.3406, *p* < 0.0001). For male cases, variables with absolute values of correlation coefficient ≥0.3 were serum albumin (*r_s_* = −0.3746, *p* < 0.0001), ECW to TBW ratio (*r_s_* = 0.3775, *p* < 0.0001), branched-chain amino acid to tyrosine ratio (*r_s_* = −0.3379, *p* = 0.0002), and serum Zn level (*r_s_* = −0.3641, *p* < 0.0001). For female cases, variables with absolute values of correlation coefficient ≥0.3 were age (*r_s_* = 0.4237, *p* < 0.0001), ECW to TBW ratio (*r_s_* = 0.5196, *p* < 0.0001), and serum Zn level (*r_s_* = −0.3214, *p* < 0.0001). In the multivariate analyses of factors linked to frailty score for males, ECW to TBW ratio (*p* = 0.0128) and SMI (*p* = 0.0223) were found to be significant, whereas serum Zn level tended to be significant (*p* = 0.0903) (Table 3). In the multivariate analyses of factors linked to frailty score for females, ECW to TBW ratio (*p* < 0.0001) and serum Zn level (*p* = 0.0406) were found to be significant (Table 4).

## 4. Discussion

As mentioned earlier, the concept of frailty has recently been extended to CLD patients. Any chronic organ dysfunction can result in physiological vulnerability [33]. However, there have been several unsolved problems regarding frailty in CLDs. The elucidation of the relationship between serum Zn level and frailty in CLDs may be of clinical importance for the better understanding of frailty in CLDs.

In our data, serum Zn level was well stratified according to the frailty status (i.e., frailty, prefrail, and robust) for all cases and for all subgroups except for non-LC cases. The multivariate analysis for frailty score revealed that serum Zn level tended to be a significant predictor in males, and it was significant in females. It should be noted that serum Zn level had already decreased, even in the prefrail stage. PA leads to a decrease in pro-inflammatory cytokines and an increase in anti-inflammatory cytokines, muscle hypertrophy, regeneration, and increased glucose uptake [34]. The differences of the serum Zn level between PA decline and PA non-decline reached significance for all analyses. These results suggest that serum Zn level can be a useful marker for frailty in CLDs. A previous study indicated that higher Cu to Zn ratio was linked with musculoskeletal health decline and frailty in the elderly [35]. Zn deficiency can be linked to numerous clinical symptoms, which may be attributed to our current results [16,17,18,19,20,21,22]. LC is often complicated by protein–energy malnutrition and also contributes to the numerous consequences of frailty syndrome [36]. The median serum Zn levels in our LC patients with frailty, prefrail, and robust were 49 μg/dL, 65.4 μg/dL, and 69.55 μg/dL, respectively, whereas those in our non-LC patients were 69.2 μg/dL, 75.5 μg/dL, and 80.65 μg/dL, respectively. Non-LC patients had much higher serum Zn levels compared to LC patients. These distinct differences of serum Zn level between LC and non-LC may account for the reason behind the non-significant stratification of serum Zn level according to the frailty status in non-LC patients.

Insulin-like growth factor-1 (IGF-1) is secreted from the liver, but is also secreted from the muscles during training, and acts on the muscles themselves, promoting the growth of muscle satellite cells that are required for muscle fiber regeneration [37,38]. IGF-1 has been demonstrated to contribute to the increase of muscle mass [37,38]. There is evidence indicating that Zn, along with appropriate protein and calorie intake, could significantly affect IGF-1 levels [39]. Serum zinc level can be associated with hormones of the growth hormone (GH)-IGF1 system [40]. In patients with CLDs, decreased serum Zn level can lead to the imbalance of hormones of the GH-IGF1 system, which may eventually result in frailty.

In our results, the absolute values of correlation coefficient between frailty score and ECW to TBW ratio were the highest for all cases (*r_s_* = 0.4386, *p* < 0.0001), male (*r_s_* = 0.3775, *p* < 0.0001) and female (*r_s_* = 0.5196, *p* < 0.0001). ECW to TBW ratio implies extracellular fluid status (i.e., water homeostasis) in the whole body and liver functional reserve [41]. Water homeostasis in CLDs may also lead to physical functional decline and cognitive decline, which can be linked to our current results [42,43,44,45]. On the other hand, body mass index did not have close correlation with frailty score for all cases (*r_s_* = −0.0768, *p* = 0.1964), male (*r_s_* = −0.1444, *p* = 0.1067), and female (*r_s_* = −0.0212, *p* = 0.7910). Frailty may not be profoundly affected by fat metabolism. Higher ECW to TBW ratio may affect body mass index due to water retention. However, it was notable that 139 (48.8%) out of 285 patients reported fatigue in our data. The long-term suffering caused by CLD itself may be one of possible reasons [46]. Frailty may be influenced by chronic disease burden. Frailty is originally defined as a geriatric syndrome, however, in our 128 patients aged less than 65 years, 71 patients (55.5%) had frailty or prefrail. Clinicians should be aware that younger age itself cannot deny the presence of frailty in CLDs.

Our study had some important limitations that should be mentioned. Firstly, this was a retrospective cross-sectional observational study that only enrolled patients in a single hospital. Secondly, our patients were of Japanese ethnicity, and therefore the results may not be generalizable to other ethnic groups. Further studies on other ethnicities are necessary to confirm and expand this research’s adaptation to other ethnicities. Thirdly, patients with large ascites who may have been involved in WS decline, GS decrease, or low PA were excluded due to the limits of BIA, due to the possibility of bias. Fourthly, details of use of diuretics potentially related to serum Zn level decrease were unclear in this analysis. Fifthly, plasma Zn level, which may be more representative to the actual blood pathophysiology status of the patient, was not tested in this study, also creating bias [47]. Finally, owing to the cross-sectional nature of our study, the direction of association between baseline serum Zn level and frailty was unclear. Interpretation with caution to our data is needed. Our study results nevertheless implied that serum Zn level and frailty in CLDs had close association. In conclusion, decreased serum Zn level in patients with CLDs can be closely associated with the presence of frailty. Close monitoring of serum Zn level in CLD patients with frailty or prefrail and optimal management will be needed.

## Figures and Tables

**Table 1:** Baseline characteristics (*n* = 285).

Variables	All Cases (*n* = 285)
Age (years)	66 (55.5, 72)
Gender, male/female	126/159
Liver disease etiology HCV-related/HBV-related/HBV- and HCV-related/NBNC-related	157/49/7/72
Presence of frailty, yes/no	38/247
Presence of LC, yes/no	107/178
Body mass index (kg/m^2^)	22.9 (20.5, 25.75)
Walking speed (m/s)	1.314 (1.111, 1.447)
Grip strength (kg), male	33.0 (27.25, 37.075)
Grip strength (kg), female	20.8 (17.7, 24.7)
SMI (kg/m^2^), male	7.435 (6.82, 8.0)
SMI (kg/m^2^), female	5.94 (5.49, 6.48)
ECW to TBW ratio	0.39 (0.384, 0.3955)
Total bilirubin (mg/dL)	0.8 (0.6, 1.1)
Serum albumin (g/dL)	4.2 (3.9, 4.5)
Prothrombin time (%)	90.5 (79.6, 98.2)
Platelet count (×10^4^/mm^3^)	17.3 (12.35, 21.15)
AST (IU/L)	24 (19, 34)
ALT (IU/L)	18 (13, 30.5)
Total cholesterol (mg/dL)	182 (150, 214)
Triglyceride (mg/dL)	85 (63.5, 116.5)
HbA1c (NGSP)	5.7 (5.4, 6.0)
eGFR (ml/min/1.73 m^2^)	81 (67.5, 94.5)
Serum sodium (mmol/L)	140 (139, 141)
Serum zinc (μg/dL)	73 (64.25, 82)
Serum ammonia (μg/dL)	20 (30, 39)
Branched-chain amino acid to tyrosine ratio	5.64 (4.17, 6.795)

Data are expressed as number or median value (interquartile range). HCV: hepatitis C virus, HBV: hepatitis B virus, NBNC: non-B and non-C, LC: liver cirrhosis, SMI: skeletal muscle index, ECW: extracellular water, TBW: total body water, AST: aspartate aminotransferase, ALT: alanine aminotransferase, NGSP: National Glycohemoglobin Standardization Program, eGFR: estimated glomerular filtration rate.

**Table 2:** Correlation between frailty score (0–5) and baseline numerical variables.

	All Cases		Male		Female	
	*r* *_s_*	*p*-Value	*r* *_s_*	*p*-Value	*r* *_s_*	*p*-Value
Age	0.3570	<0.0001	0.2740	0.0019	0.4237	<0.0001
Body mass index	−0.0768	0.1964	−0.1444	0.1067	−0.0212	0.7910
Total bilirubin	0.0009	0.9874	−0.0736	0.4128	0.0528	0.5088
Serum albumin	−0.3212	<0.0001	−0.3746	<0.0001	−0.2774	0.0004
Prothrombin time	−0.1045	0.0783	−0.1161	0.1954	−0.0860	0.2869
Platelet count	−0.1933	0.0010	−0.1396	0.1191	−0.2290	0.0037
AST	0.1013	0.0879	0.1112	0.2153	0.0908	0.2551
ALT	0.0142	0.8118	−0.0813	0.3654	0.0949	0.2342
Total cholesterol	−0.1813	0.0021	−0.0720	0.4231	−0.2518	0.0014
Triglyceride	0.0467	0.4326	−0.0630	0.4836	0.1227	0.1233
HbA1c (NGSP)	0.0047	0.9375	0.0423	0.6378	−0.0191	0.8114
eGFR	−0.0409	0.4921	−0.0306	0.7338	−0.0495	0.5341
SMI	NA	NA	−0.2155	0.0164	−0.2345	0.0029
ECW to TBW ratio	0.4386	<0.0001	0.3775	<0.0001	0.5196	<0.0001
BTR	−0.2374	<0.0001	−0.3379	0.0002	−0.1514	0.0652
Serum zinc	−0.3406	<0.0001	−0.3641	<0.0001	−0.3214	<0.0001
Serum ammonia	0.0632	0.3007	0.2050	0.0259	0.0374	0.6471

AST: aspartate aminotransferase, ALT: alanine aminotransferase, NGSP: National Glycohemoglobin Standardization Program, eGFR: estimated glomerular filtration rate, SMI: skeletal muscle index, ECW: extracellular water, TBW: total body water, BTR: branched-chain amino acid to tyrosine ratio.

**Table 3:** Multivariate analyses of factors linked to frailty score for males.

	Estimates	Standard Error	*p*-Value
Age	−0.00472	0.0110	0.6693
Serum albumin	−0.216	0.272	0.4289
SMI	−0.238	0.102	0.0223
BTR	−0.00292	0.0648	0.6536
Serum zinc	−0.00847	0.00821	0.0903
Serum ammonia	0.00494	0.00361	0.1738
ECW to TBW ratio	21.607	14.091	0.0128

SMI: skeletal muscle index, BTR: branched-chain amino acid to tyrosine ratio, ECW: extracellular water, TBW: total body water.

**Table 4:** Multivariate analyses of factors linked to frailty score for females.

	Estimates	Standard Error	*p*-Value
Age	0.00672	0.00773	0.3860
Serum albumin	−0.109	0.189	0.5648
SMI	−0.150	0.105	0.1567
Platelet count	0.0135	0.0117	0.2509
Serum zinc	−0.0131	0.00635	0.0406
Total cholesterol	−0.00237	0.00182	0.1939
ECW to TBW ratio	61.248	11.351	<0.0001

SMI: skeletal muscle index, ECW: extracellular water, TBW: total body water.

## Abbreviations

PA	physical activity
BW	body weight
WS	walking speed
CLD	chronic liver disease
Zn	zinc
LC	liver cirrhosis
GS	grip strength
SMI	skeletal muscle index
BIA	bioimpedance analysis
IQR	interquartile range
ECW	extracellular water
TBW	total body water
IGF-1	insulin-like growth factor-1
GH	growth hormone
